# A flexible physical protection process for lignin extraction

**DOI:** 10.1016/j.isci.2023.107507

**Published:** 2023-07-28

**Authors:** Maria Karlsson, Martin Lawoko

**Affiliations:** 1 Wallenberg Wood Science Center, Department of Fiber and Polymer Technology, School of Chemistry, Biotechnology and Health, KTH Royal Institute of Technology, Teknikringen 56-58, 100 44 Stockholm, Sweden; 2 Division of Wood Chemistry and Pulp Technology, Department of Fiber and Polymer Technology, School of Chemistry, Biotechnology and Health, KTH Royal Institute of Technology, Teknikringen 56-58, 100 44 Stockholm, Sweden

**Keywords:** Chemistry, Biotechnology, Biomass

## Abstract

Research on lignin valorization has gained ground, driven by its potential to replace fossil-based phenolics in bio-based applications. Technical lignins are structurally complex and still poorly characterized, prompting the need for novel extraction processes for lignin of high analytical quality. In this context, a two-step cyclic extraction process for lignin was contrasted with a one-step cyclic extraction. The latter was shown to preserve the native structure of the spruce lignin product better and improved the yields of both the extracted lignin and residual fiber fraction. The application of the one-step cyclic extraction process to birchwood resulted in a similar protection of the lignin structure. Overall, a flexible physical protection (FPP) process for extraction of lignin with an abundance of native bonds is presented. The lignin product has a high abundance of ether bonds and hydroxyl functionalities, which are of interest in biochemical, polymer, and material applications.

## Highlights

• A flexible physical protection (FPP) process for native lignin extraction is developed
• The FPP process is a green process and is applicable to different wood species
• The lignin has a bright color, is odorless, and has high purity
• The lignin properties can be user-case tailored by FPP process parameters

## Graphical abstract

**Figure:**
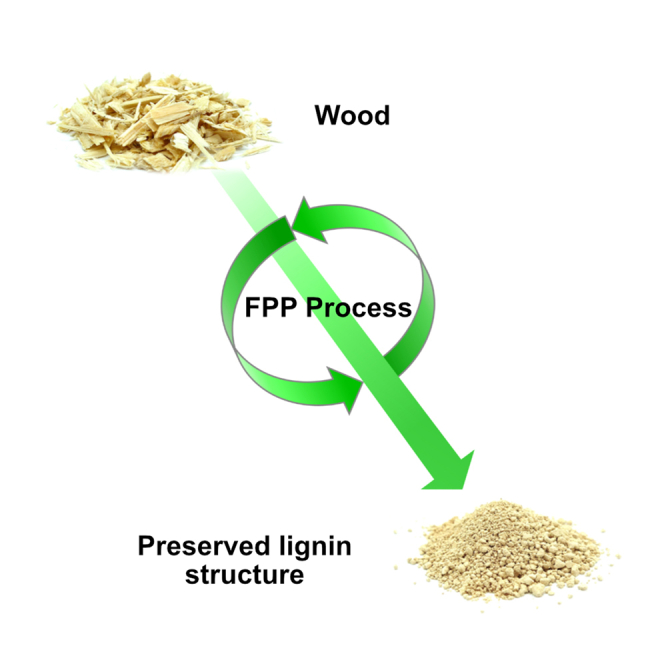

## Introduction

Lignin is the most abundant naturally occurring aromatic.^1^ Lignin accounts for 15%–30% of the biomass weight, depending on the species.^2^^,^^3^ In the quest for a sustainable production and consumption, research on the use of lignin as raw material to replace fossil-based equivalents in different applications has gained momentum and includes areas such as biomaterials, biochemicals, and biomedical applications.^4^^,^^5^^,^^6^^,^^7^^,^^8^ The raw materials being investigated include available technical lignins and lignin extracted using emerging biorefinery protocols.^9^ Traditional pulping processes are water based and performed at either alkali or acidic conditions, often with sulfur-containing chemicals. The lignin retrieved from these processes is commonly referred to as technical lignin. Technical lignin is complex in structure due to severe modification of native lignin structures during the process. The complexity in structure imposes analytical challenges. As a result, the structure of technical lignin is to date still poorly characterized.^10^^,^^11^ This presents a major drawback in using technical lignin in application studies, where well-characterized precursors are required to understand, predict, and control reactivity, as well as gain fundamental insights on structure-property relationships in synthesized materials.

Organosolv processes have been investigated; however, they have not been applied industrially as a pulping process. An organosolv process that obtained pilot scale status is the "Alcell" process.^12^ In this process lignin is extracted at high temperature with aqueous ethanol, often with an inorganic acid as catalyst.^13^^,^^14^ The acidic conditions also promote lignin condensation reactions.^15^ More specifically, at the low pH and high extraction temperature, benzylic cations are formed and subsequently react with the electron-rich aromatic ring in condensation reactions.^16^ Condensation reactions complicate lignin analytics due to enhancement in the structural heterogeneity. This is manifested in the low levels of structural assignments achieved for technical lignin such as kraft lignin^10^^,^^11^ and organosolv lignin.^17^

To minimize the occurrence of condensation reactions, chemical and physical protection strategies have been evaluated.^18^ Chemical protection strategies apply chemical additives to protect the lignin structure through selective capping of the carbocation-prone sites on the lignin polymer. One example is the aldehyde-assisted fractionation (AAF), where aldehydes are used to stabilize the lignin and prevent the formation of stable C-C linkages.^19^^,^^20^^,^^21^ An example adopting the physical protection principle is the flow-through concept, where a continuous flow extraction is performed to obtain lignin with a protected structure.^22^ Physical protection can also be achieved by a cyclic extraction approach, reported in our previous work.^23^^,^^24^ In the cyclic extraction method, the lignin is extracted in short cycles to obtain a lignin product with preserved structure and high analytical quality.^23^ In the interest of an integrated biorefinery concept, the cyclic extraction of lignin presided a hydrothermal extraction of the wood hemicelluloses^23^^,^^24^ intended to additionally provide a potentially useful stream of hemicelluloses. Furthermore, the potential to fine-tune lignin properties was shown in a subsequent chemometric study.^24^

In the present work, we sought to investigate the potential to tailor the properties of the residual fibers through retention of hemicelluloses in the fibers. Accordingly, a one-step cyclic extraction of lignin is herein investigated, and the universality of its positive effects in terms of lignin product quality and yield is demonstrated through its application on softwood and hardwood species as raw materials.

## Results and discussion

### Lignin analytics

**Figure 1:**
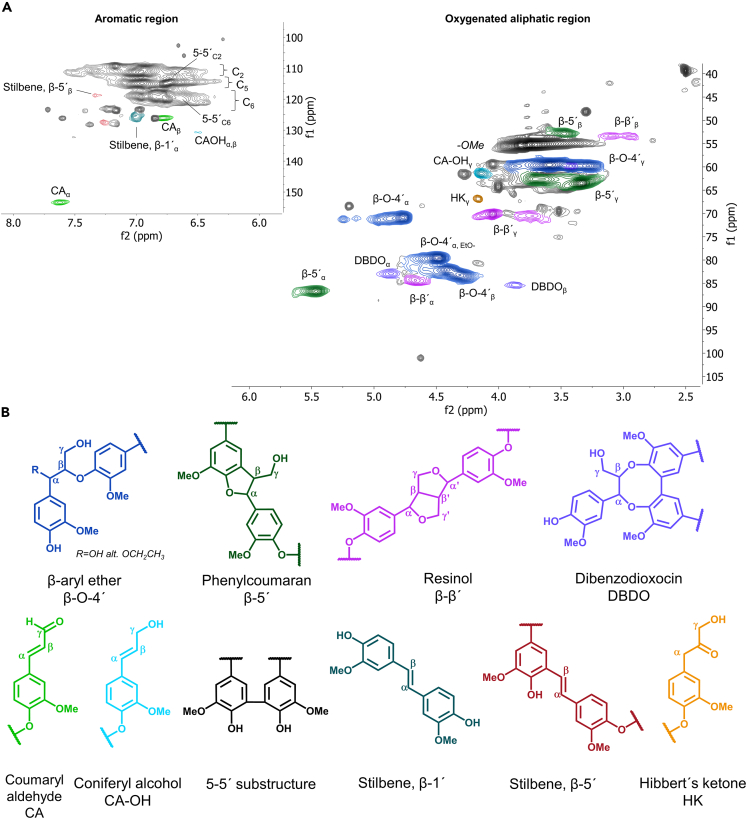
HSQC NMR spectrum and interunit linkages for spruce cyclic extracted lignin HSQC NMR spectra of 9C, 140°C spruce lignin in DMSO-d6 (A) and assigned interunit linkages commonly reported for spruce, both native and process-altered structures (B).

The interunit linkages, characterized by heteronuclear single quantum coherence (HSQC) nuclear magnetic resonance (NMR), showed an abundance of native structures (Figures S1–S5). In Figure 1, the assigned HSQC spectrum of lignin extracted using 9 cycles (C) at 140°C and interunits are shown. The spectrum displays typical features of signals assigned to native lignin interunits. The interunit linkages were semi-quantified using the aromatic carbon-2 and attached hydrogen (C2-H) signal as an internal reference for the quantification^25^ (Table 1). The selected extraction conditions enabled comparison between the two-step extraction from our previous work and the one-step extraction in this work. Moreover, extraction conditions presumed to be suitable for the extraction of lignin with maximally preserved lignin structure were included. As a measure for the protection efficiency, the content of β-*O*-4' bonds was used because these bonds are more prone to react at the extraction conditions than the other common bonds in lignin. Therefore, the higher the content of β-*O*-4′ bonds, the more efficient the preservation of native lignin bonds. In our previous work,^24^ a chemometric study identified that conditions of 140°C and 1.5% acid preserved the β-*O*-4' bonds best. Hence these conditions were also included in the present study, in combination with 4C and 9C, and studied by HSQC to reveal β-*O*-4′ contents of 53% and 54%, respectively (Table 1). These results highlighted a superior preservation of native lignin structure in lignin extracted by the one-step approach when contrasted with the typically studied lignins obtained by other methods now discussed.

**Table 1:** Quantification of interunit linkages by HSQC NMR for spruce lignin obtained using the cyclic extraction method at different conditions

Spruce lignin	β-O-4′_α_	DBDO_β_	β-5′_α_	β- β′_α_	Stilbene, β-1′_α_	Stilbene, β-5′_β_	CA_α_	HK_γ_^a^
***per 100 Ar***								

4C, 140°C	53	1.6	13	2.2	6.3	1.9	2.7	0.75
4C, 160°C^b^	42	0.5	12	2.1	3.4	3.3	2.6	1.6
9C, 140°C	54	2.3	14	2.2	4.0	1.1	2.7	0.75
15C, 160°C	31	1.6	10	2.1	4.8	1.4	1.8	1.3

DBDO, Dibenzodioxocin; CA, Coumaryl aldehyde.

The interunit linkages are quantified per 100 Ar using diagnostic chemical shifts, Table S1.

a K=Hibbert’s ketone.

b 4 cycle extraction immediately after 4 cycle extraction at 140°C.

For instance, milled wood lignin, extracted from ball-milled wood, is broadly used as a model for “native” lignin. The β-*O*-4′ content of milled wood lignin (softwood) is in the proximity of 35%.^26^ In contrast, based on lignification theory and model studies, it is proposed that native spruce lignin has a β-*O*-4′ content of around 60%, and the respective amount for the hardwood birch is in the proximity of 80%.^27^ Furthermore, typical β-*O*-4′ contents for lignin derived from Alcell organosolv processes using a batch extraction are below 10%.^23^^,^^28^ As demonstrated here, by using the optimized cyclic extraction method, lignin with a β-*O*-4′ content close to that suggested for native lignin based on lignification theory^27^ was obtained. Since the HSQC NMR analysis of lignin is, at best, semi-quantitative, verification through quantitative ^13^C NMR analysis was required. The ^13^C NMR analysis was performed for the 4C, 140°C and 9C, 140°C lignin and surprisingly revealed only negligible differences in the β-*O*-4′ contents when compared with the HSQC analysis (53% and 52%, respectively) (Figures S10 and S11 and Table 1).

**Figure 2:**
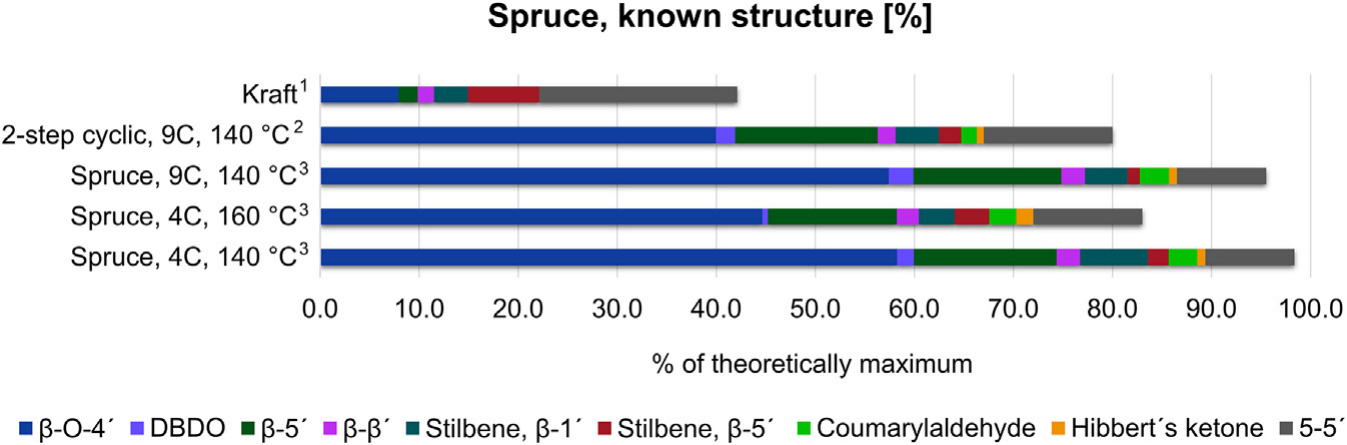
Quantification of commonly reported interunit linkages with both native and process-altered structures for spruce The values are contrasted with benchmark lignin, by kraft lignin and lignin obtained by the 2-step cyclic process. The amount of 5-5′ interunit linkages was estimated from the HSQC 5-5′C2 signal and in accordance with previously reported estimated values.^24^ The content of interunit linkages measured by NMR was corrected using the DPn of the lignin fraction, Table S5. ^1^ Kraft lignin derived from the LignoBoost process. The results are based on previous work.^24^ ^2^ Lignin retrieved from the 2-step cyclic extraction presented in previous work.^23^^,^^24^ ^3^ The one-step cyclic extraction method performed in this study.

A more accurate content of the interunit linkages was achieved for the two samples by taking into consideration the degree of polymerization (DP_n_) obtained from the size exclusion chromatography (SEC) analysis. The results are shown in Figure 2. As comparative benchmarks, kraft lignin and the lignin obtained through the 2-step cyclic extraction approach are included in the Figure. Interestingly, for the one-step process, the assignment of more than 95% of the lignin structure was achieved for the 140°C extracted lignin. Moreover, assignment of 85% of the lignin structure was achieved for the 160°C extracted lignin. These values can be contrasted with kraft lignin, where roughly 40% of the structure was analyzed. The cyclic extraction approach is therefore beneficial for the recovery of lignin with well-defined chemical properties.

The β-*O*-4′ content for lignin obtained by the 2-step cyclic extraction process performed at 140°C was reported at 36%.^24^ At a higher temperature, i.e., 160°C, the β-*O*-4′ content was about 30%.^23^ In contrast, this study shows a β-*O*-4' content in the proximity of 54% for the one-step cyclic extraction of spruce lignin, demonstrating a significantly better physical protection of the lignin compared to the 2-step approach used in the previous work. This difference can be explained by the chemistry events during the hydrothermal hemicellulose extraction preceding the lignin extraction in the 2-step process. Such events include auto hydrolysis leading to cleavage of some β-*O*-4′ bonds and the occurrence of lignin condensation reactions.^23^^,^^29^^,^^30^ Considering that the hydrothermal pretreatment was performed in batch mode (as opposed to cyclic mode) and for a 2-h duration at 160°C, the likelihood of the occurrence of condensations reactions is high. It is also reported that such condensed lignin, if present in the process liquors, are re-deposited onto the fibers.^31^ Such re-deposited lignin will likely constitute part of the extracted lignin in the subsequent cyclic extraction stage of the 2-step process. On the contrary, the direct extraction of lignin adopting the cyclic approach, i.e., the one-step cyclic extraction, circumvents the structural changes that otherwise occur when the hydrothermal pretreatment is performed and consequently yields a structurally less-modified lignin.

The β-*O*-4′ appears to be both hydroxylated and etherified on the C_α_ position, which is in accordance with previous reports.^32^ The benzylic etherification is explained by the end capping of reactive benzylic cation by ethanol.^23^^,^^24^

**Figure 3:**
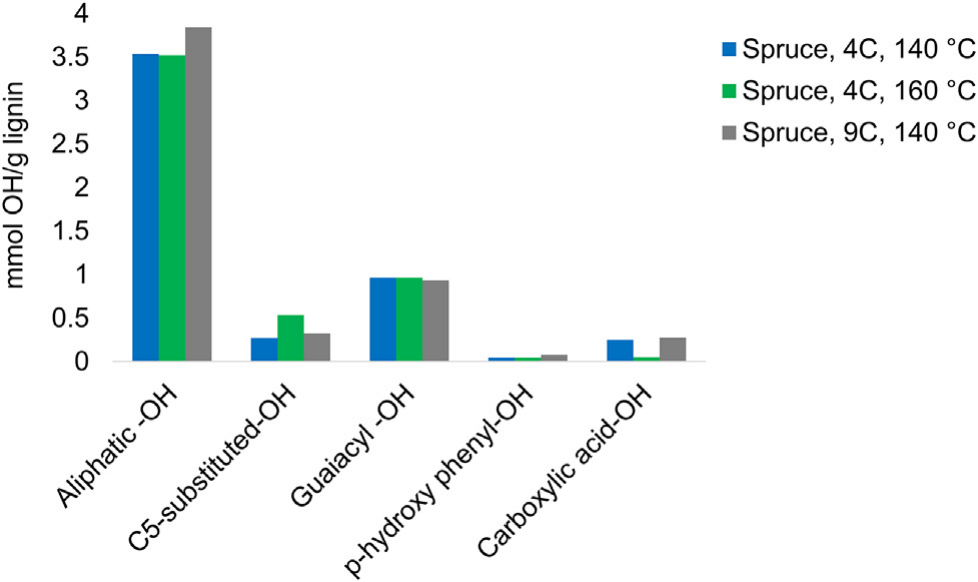
Hydroxyl (OH) functionalities of lignin derived at different numbers of cycles and temperatures

For the same number of cycles (4C), it is observed that the β-*O*-4' content dropped from 53% to 42% when the extraction temperature was changed from 140°C to 160°C (Table 1). The temperature, therefore, had an effect on the β-*O*-4′ content. The relation between temperature and β-*O*-4′ content has been shown in our earlier work.^24^ The decrease in β-*O*-4′ with the increase in extraction temperature may be partially explained by acidolysis reactions that yield Hibberts ketones.^33^^,^^34^ This is supported by the increased content of Hibberts ketones at higher temperatures (Figures 1 and 3). The lignin fractions had high purity with trace signals originating from carbohydrates (Figures S1–S5, estimated <1%).

The hydroxyl (OH) functionalities of the lignins were studied by phosphorus-31 (31P) NMR (Figures S6–S9 and Table S3). The results showed a higher content of aliphatic OHs in the cyclic extracted lignin relative to Alcell-based lignin (Table S4). This difference is explained by the preservation of the native lignin structure configuration in the cyclic extracted lignin, where Cα and Cγ on the aliphatic side chain are to a large extent hydroxylated.^35^ However, as noted earlier, the Cα positions of the cyclic extracted lignin were partially etherified and partially hydroxylated, meaning that a higher content of aliphatic-OH may be expected in the native lignin.

An increase in the content of C5-substituted phenolic OH is observed with increasing temperature (Figure 3). This is likely a consequence of the enhanced hydrolysis of ether bonds in etherified 5-5′ linkages which include dibenzodioxocins and others that may constitute branching points in native lignin.^36^ A comparison of the different OH contents with those obtained from Alcell organosolv process at batch conditions (Table S4) shows that the Alcell lignin has an aliphatic OH content less than half that of the cyclic extracted lignins (cf. 1.4 mmol/g with 3.5 mmol/g) but more than double the phenolic OH content of the cyclic lignins (Table S4). This is consistent with the enhanced hydrolysis of aryl ethers and occurrence of condensation and elimination reactions when operating the extraction in batch rather than cyclic mode.

To summarize this part, the one-step cyclic extraction, when contrasted with the previously reported two-step process, shows similarities with respect to lignin purity and better preservation of lignin structure, explained by the exclusion of the hydrothermal extraction step in which some structural changes in lignin occur^23^. The physical protection principle intrinsic to the cyclic extraction was therefore maintained.

### Investigating the universal application of the physical protection concept

**Figure 4:**
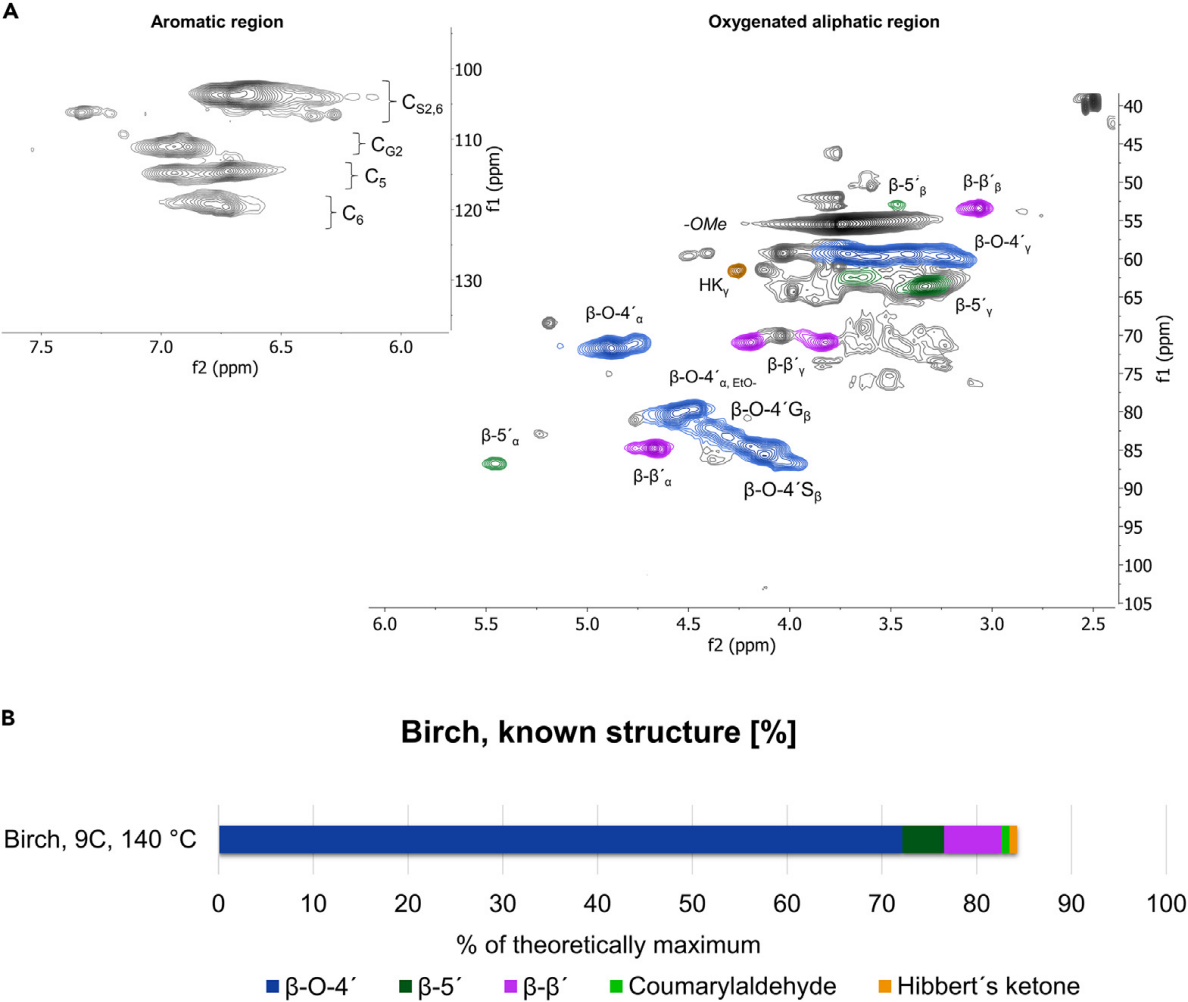
HSQC NMR spectrum for cyclic extracted birch lignin and the content of inter-unit linkages HSQC NMR spectra from 9C, 140°C birch lignin in DMSO-d6 (A) together with quantified interunit linkages (B). The content of interunit linkages is reported as the percentages adjusted toward the DPn of the lignin fraction, Table S5.

The universality of the physical protection concept was investigated by subjecting birchwood to the similar process as softwood spruce. More specifically, the extraction method applied for spruce (9C, 140°C) was used on the hardwood birch specie. The HSQC spectra are shown in Figure 4A. A β-*O*-4' content of 67% was determined (Table S2), and the β-*O*-4′ appears to be both hydroxylated and etherified on the Cα position, similar to the spruce lignin (Figure S5). The theoretical content of interunit linkages was estimated more accurately (Figure 4B), in a similar manner to the spruce lignin, using the DPn calculated from the SEC data (Table S5).

About 85% of the birch lignin structure was assigned (Figure 4), which was quite significantly lower than that of spruce lignin determined at 95%. This difference could be explained by a cluster of signals in the CH region 70–75 ppm/3–3.7 ppm being detected but not assigned. Signals in this region are commonly assigned to carbohydrates. However, the absence of signals attributed to the anomeric carbons in carbohydrates, which would appear in the region 97–105 ppm/4–5 ppm, suggests the unidentified clusters were likely lignin-related benzyl alkyl ethers. The relative intensities of the unassigned signals are higher in the birch lignin (Figure 4) than in the spruce lignin (Figure 1A), suggesting that benzyl alkyl ethers may be more abundant in birch lignin than in spruce lignin.

The 31P NMR spectrum of the extracted birch lignin is shown in Figure S9. Typically, the analysis of hydroxyls in hardwood lignins by 31P NMR is complicated by overlap of signals from syringyl- with signals from C5-condensed phenolics. For this reason, the detailed studies of the types of hydroxyls in hardwoods and the comparison with the softwood lignins are compromised. Nevertheless, a simplified comparison of the total OH content was made and suggests the contents are quite similar (5.2–5.4 mmol/g lignin) for the 9C, 140°C lignins.

### Fractionation-SEC results

**Figure 5:**
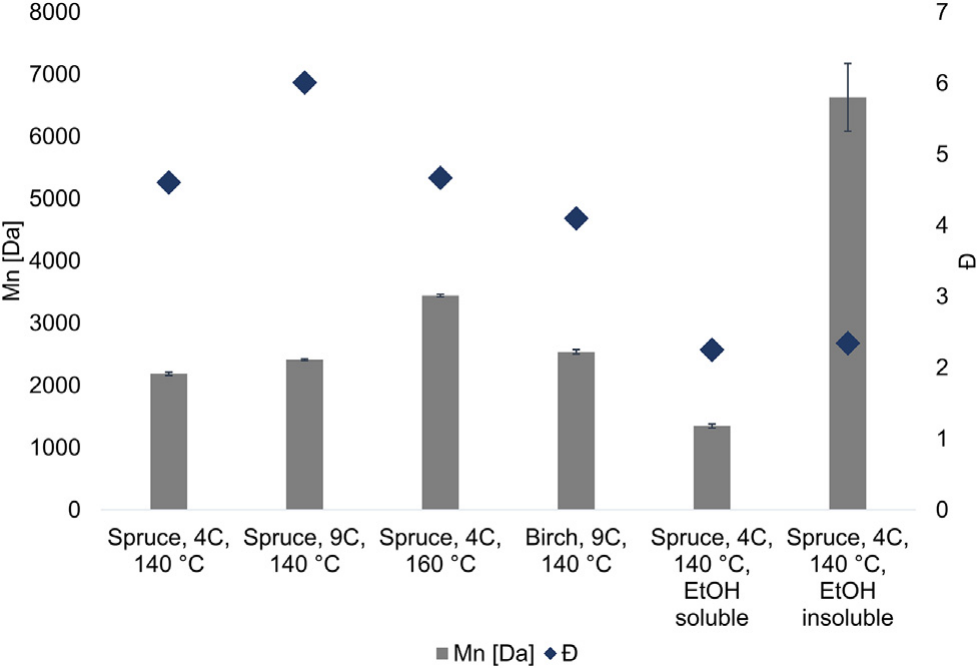
The molecular weight distribution (Mn) and dispersity (Đ) of the lignin fractions Error bars are shown in the figure and indicate error margin in the range 20-30 dalton for all entries except for the ethanol insoluble fraction which shows a margin of 550 dalton.

The average molecular weight distribution (Mn) and dispersity (Đ) of lignin determined by SEC are estimations due to the lack of representative standards for lignin. The values reported here may therefore be viewed as relative and mainly of value for comparative studies. The Mn of the spruce lignins 4C, 140°C and 9C, 140°C were 2,200 and 2,400 Da, respectively (Figure 5 and Table S5). The 9C, 140°C birch lignin had an Mn of 2,500 Da, which was in proximity to the value for the spruce lignin obtained using the same extraction conditions. The Mn, however, increased with the increased temperature for spruce (Figure 5). This is likely due to the increased solubility of higher-molecular-weight lignin, or possibly, the minor occurrence of intermolecular condensation reactions during the extraction.

To reduce the size heterogeneity, the 4C, 140°C lignin was treated with absolute ethanol at ambient conditions to yield an ethanol-soluble fraction (∼60%) and an insoluble fraction (∼40%) (Figure 5). A clear improvement in Đ was observed for both the soluble and insoluble fractions, which interestingly had a similar Đ (2.3 and 2.2, respectively) (Figure 5 and Table S5). There was a significant Mn difference between the fractions, where the ethanol-soluble part had an Mn of 1,300 Da and the ethanol insoluble part had an Mn of 6,600 Da. This demonstrates that the cyclic lignin could be further refined to obtain fractions with different properties that may be suited for different applications. For instance, low-dispersity lignin with Mn in the 1,000–1,300 Da range has been used to synthesize lignin-based epoxyresins^8^ and Lignin-thiol-ene resins.^7^ Noteworthy is that the refining adopts the use of ethanol, which is also the solvent in the extraction process. This is beneficial from both process economy and environmental viewpoints since no new solvent is required; the process solvent is used to refine the crude product.

### The yield of the lignin and fiber fraction

**Figure 6:**
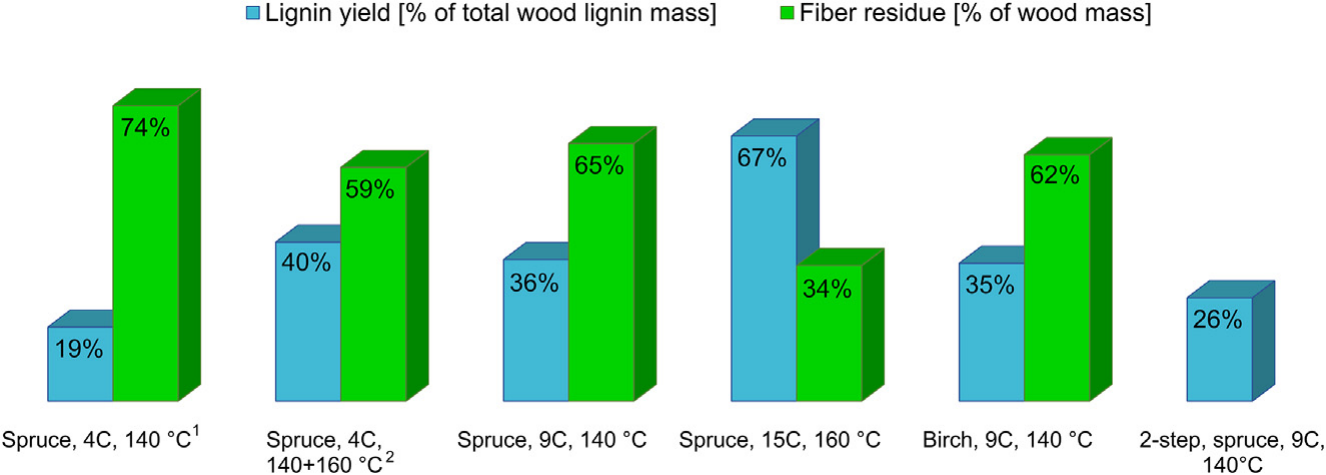
The yield of the obtained lignin using the different number of cycles and temperatures together with the yield of the fiber residues ^1^Number of replicates, n = 3: Standard deviation, lignin: ±0.21%. Standard deviation, fiber residue: ±3.0%. ^2^4 cycle extraction at 160°C, immediately after 4 cycle extraction at 140°C. Lignin yield: 4C, 140°C: 19%, 4C, 160°C: 21%.

The lignin and fiber residue yields are presented in Figure 6. The yield evaluation of the lignin and fiber residue fraction showed high repeatability with a low standard deviation (Figure 6, notes). The lignin yield is reported as a percentage of the total lignin mass in the respective wood species, and the fiber residue as a percentage of the total mass of the oven-dried original wood.

For the spruce series, the fiber yield decreased and the lignin yield increased with an increased temperature. Interestingly, the lignin and fiber yields were almost the same for spruce and birch at the same extraction condition. There was a part of the material that was not recoverable in pure organic state due to being soluble in the water phase (Figures S12 and S13). At the lower temperature of 140°C, the unrecovered material accounted for 20%–30% of the original wood mass. For the higher temperature of 160°C, this unrecovered material was even higher, 40%–50%.

A comparison of the lignin yields at the lower temperature (9C, 140°C) showed that the one-step cyclic extraction performed better than the 2-step extraction from our previous work^24^ (36% versus 26%, Figure 6). At the higher temperature (15C, 160°C), the one-step extraction performed better in lignin yield but worse in fiber yield when compared with the two-step protocol (34% versus 44%^24^). The reason for the lower fiber yields are not known, but it is evident that some events in the hydrothermal treatment step of the two-step process affected the hydrolysis and dissolution of the polysaccharides in the subsequent cyclic extraction step. One hypothesis is that the hydrothermal extraction of the bulk of the hemicelluloses may have caused a closer association of the retained polysaccharides with lignin, the latter acting as a barrier that in effect reduced the efficiency of acid hydrolysis of the polysaccharides in the subsequent cyclic extraction step. Further studies are required to gain insights into this interesting result.

### Carbohydrate analysis

The carbohydrate analysis was performed for pure milled spruce wood reference and fiber residues from 4C, 140°C and 15C, 160°C (Table 2). The 4C, 140°C sample showed a small change in the composition of sugars, reflecting a slight enrichment of glucose. This suggests enrichment of cellulose in the fiber residue. The enrichment of cellulose is further enhanced in the fiber residue of the 15C, 160°C sample. This was an indication of increased hemicellulose hydrolysis and in agreement with the yield analysis discussed earlier. The tunability of the cyclic extraction process in terms of lignin properties and the fiber residue composition was thus validated.

**Table 2:** The relative composition % of the carbohydrates in spruce wood and fiber residues (4C,140°C and 15C, 160°C)

Carbohydrate	Spruce wood	Spruce, 4C, 140°C	Spruce, 15C, 160°C^a^
**Relative composition %**			

Arabinose	1.7 ± 0.00065^a^	0.14 ± 0.0072	ND
Galactose	3.5 ± 0.043	1.3 ± 0.029	ND
Glucose	70 ± 0.070	78 ± 0.51	97 ± 0.15
Xylose	8.2 ± 0.010	6.6 ± 0.10	1.1 ± 0.056
Mannose	16 ± 0.056	13 ± 0.34	2.2 ± 0.098
GalA	0.86 ± 0.034	0.68 ± 0.047	ND
GlcA	0.094 ± 0.0045	0.05 ± 0.0015	ND

ND, not detected.

a Number of replicates, n = 2.

**Figure 7:**
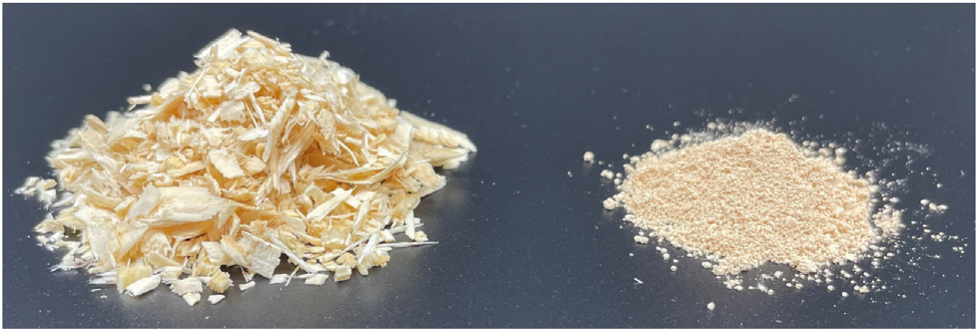
Sawdust from spruce wood shown beside lignin extracted using the cyclic extraction method (9C, 140°C) The color illustrated in the figure is also representative of 4C, 140°C and 4C+4C, 140°C+160°C lignin.

The colors of the extracted spruce lignin and the fiber residues were similar to the color of native spruce wood (Figure 7), which may reflect the preservation of native lignin structure. Using the cyclic extraction concept, only a part of the solvent is exchanged during the extraction.^23^^,^^24^ In principle, the dissolved lignin is less exposed to the extraction conditions, in effect minimizing structural changes.^23^ It is also likely that as the concentration of solutes is kept low through displacement with fresh solvent, the probability of re-polymerization decreases due to the lower incidence of molecular collisions. Additionally, for the same extraction conditions and duration, a higher yield of lignin is obtained when compared to batch extraction.

### Conclusion

Lignin with preserved native structure is particularly attractive for the production of platform monomers and biochemicals through catalytic depolymerization approaches due to the abundance of aryl ether bonds. In previous work, we developed a two-step extraction process for spruce lignin with highly preserved native structure. A hydrothermal extraction of hemicelluloses was first performed, followed by a cyclic extraction of lignin in an aqueous ethanol-based organosolv process.

In the present work, a refined one-step cyclic extraction process for lignin was investigated. It was shown that the residual fiber composition could be tailored with minimal alteration to the extracted lignin structure.

The universality of the one-step extraction was then investigated by subjecting birchwood to similar processing as spruce wood. It was shown that the physical protection principle worked equally well for birch lignin as for spruce.

The lignin analytical quality was improved by the one-step procedure when contrasted to the two-step procedure at the same extraction condition. About 95% of the lignin structure was assigned for spruce, and over 85% for the birch lignin. The aryl ether linkage (β-*O*-4′) contents of the extracted lignins were between 45% and 60% for spruce lignin and >65% for birch lignin. The lignin products had a bright beige color, typical of less-modified structures. The present work introduces a flexible physical protection process for the extraction of structurally protected lignin, where the residual fiber properties can be tailored independent of wood species.

### Limitations of the study

The focus of the present work has primarily been devoted to studying the lignin properties in relation to the extraction scheme. More detailed studies on the fiber residue and the water-soluble fractions are required and will be the subject of future work.

## STAR★Methods

### Key resources table

**Table:** 

REAGENT or RESOURCE	SOURCE	IDENTIFIER
**Chemicals, peptides, and recombinant proteins**		

Ethanol (99.8%)	VWR	Cat#20821.310; CAS: 64-17-5
Methanol (≥99.8%)	VWR	Cat#20847.307; CAS: 67-56-1
Tetrahydrofuran (HPLC grade)	VWR	Cat#28559.320; CAS: 109-99-9
Sulfuric acid (>95%, analytical grade)	Fisher Chemical	Cat#11423453; CAS: 7664-93-9
Acetic anhydride (99.7%, analytical grade)	Sigma-Aldrich	P33214; CAS: 108-24-7
Pyridine (anhydrous, 99.8%)	Sigma-Aldrich	P270970; CAS: 110-86-1
Toluene (≥99.5%)	Sigma-Aldrich	P179418; CAS: 108-88-3
Endo-N-hydroxy-5-norbornene-2,3-dicarboximide (eHNDI; 97%)	Sigma-Aldrich	P226378; CAS: 21715-90-2
chromium(III) acetylacetonate (Cr(acac3); 99.99%)	Sigma-Aldrich	P574082; CAS: 21679-31-2
2-chloro-4,4,5,5-tetramethyl-1,3,2-dioxaphospholane (Cl-TMDP; 95%)	Sigma-Aldrich	P447536; CAS: 14812-59-0
[D6]DMSO (99.9 at.% D)	Sigma-Aldrich	P151874; CAS: 2206-27-1
*N,N*-dimethylformamide (anhydrous, 99.8%)	Sigma-Aldrich	P227056; CAS: 68-12-2
CDCl3 (≥99.8 at. % D)	Sigma-Aldrich	P151823; CAS: 865-49-6
Milli-Q water (Q-POD, Millipak 0.22 μm filter)	Millipore	N/A
Wood chips (Norway spruce and birch)	N/A	N/A

**Software and algorithms**		

MestReNova (v.9.0.0)	Mestrelab Research	https://mestrelab.com
Chromeleon (v. 7.2.10)	Thermo Fisher Scientific	https://www.thermofisher.com
Chromeleon (v.7.1)	Thermo Fisher Scientific	https://www.thermofisher.com
Empower 3 (build 3471)	Waters	https://www.waters.com

### Resource availability

#### Lead contact

Further information and requests for resources and reagents should be directed to and will be fulfilled by the lead contact, Martin Lawoko (lawoko@kth.se).

#### Material availability

The generated lignin in this study can be made available on request, but we may require a payment and/or a completed Materials Transfer Agreement if there is potential for commercial application.

### Method details

#### Refined cyclic extraction process for lignin

Building on our previous work, we sought to investigate a refined method to extract lignin with preserved native linkages. Accordingly, a one-step cyclic extraction protocol was studied and contrasted with the previously published two-step protocol.^23^ The different streams from the two-step and one-step cyclic extraction process are illustrated in the figure below.

**Figure:**
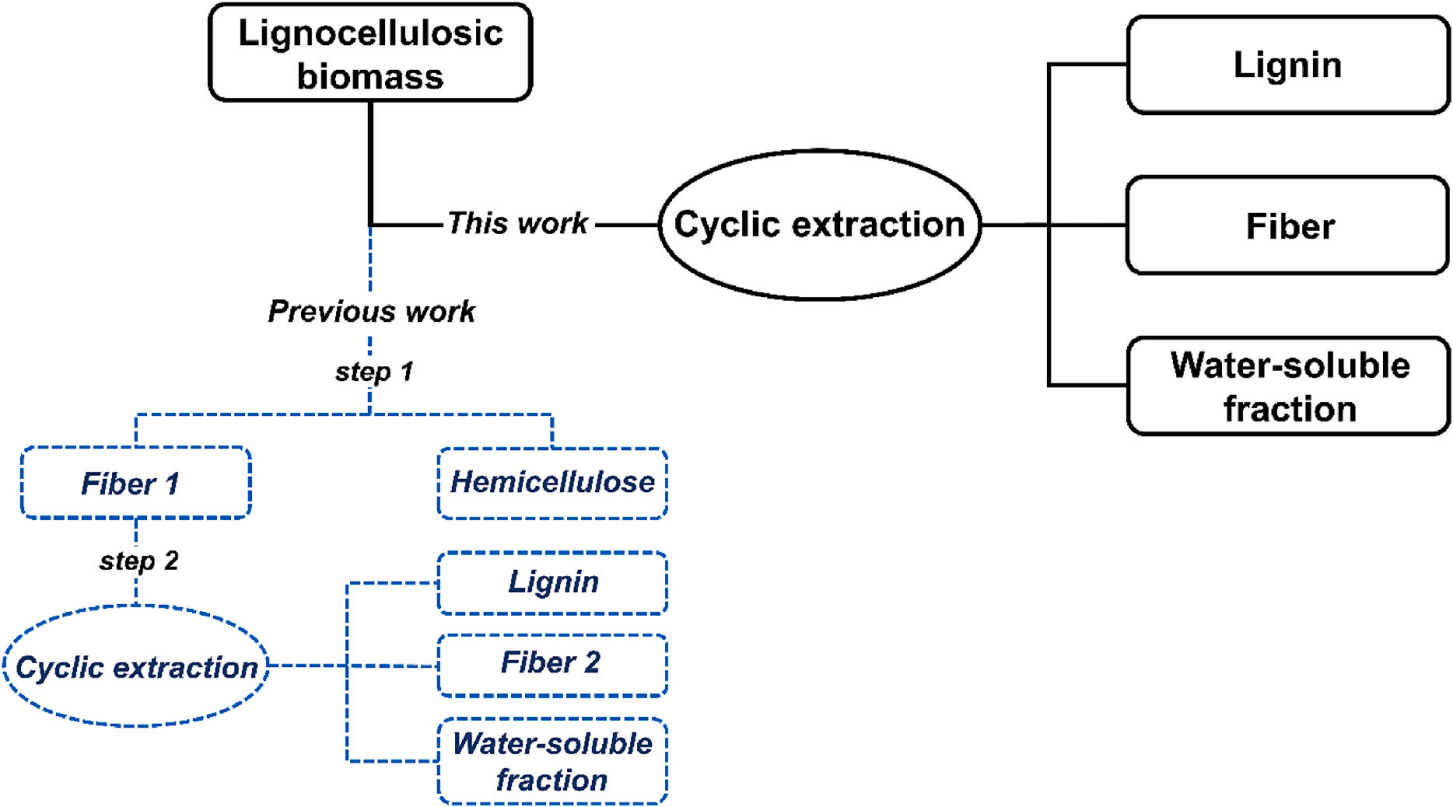
Illustration of the different streams from the cyclic extraction process used in previous work and in the present study.

#### Extraction of lignin

The wood chips were milled to a size of 40 mesh using a Wiley mini mill (3383L70, Thomas Scientific, Swedesboro, USA). The extractions were performed using an Accelerated Solvent Extractor 350 instrument (Thermo Fisher Scientific, Sunnyvale, CA, USA). A 66 ml Dionium zirconium extraction cell (Thermo Fisher Scientific, Sunnyvale, CA, USA) equipped with glass fiber filters specially designed for the extraction cell was used in the extraction. The extract was collected in 250 ml bottles. The instrument was controlled using the Chromeleon 7.2.10 software (Thermo Fisher Scientific, Sunnyvale, CA, USA). The lignin was filtered using a glass fiber filter (1.6 μm, 70 mm) purchased from VWR.

For all experiments, 9.281 g oven-dry based milled wood was impregnated with 60 ml using the solvent system of ethanol: water (70: 30 v/v) with an amount of 1.5 wt% sulfuric acid for 3 hours at ambient temperature in a closed container. The impregnated wood was placed in a 66 ml Dionium zirconium cell between the inserted glass fiber filters. The same solvent composition as for the impregnation was used for all the extractions. For all extractions, the start volume was approximately 70 ml, measured at atmospheric pressure. The extraction was performed using the ASE 350 instrument with a solvent exchange of approximately 20% of the start volume in every cycle. The number of cycles was 4 or 9 for the different experiments, with a cycle time of 5 minutes. After the last cycle, the residue in the extraction cell was purged with N_2_ for 90 s to remove the main part of the extract in the cell. The extraction was performed at a pressure of approximately 110 bar. The temperature of 140°C and 160°C was used for different extraction entries. The dissolved lignin was precipitated in the water phase by evaporation of the ethanol under reduced pressure using a vacuum rotary evaporator. A corresponding amount of water was added to the extract before the evaporation of ethanol to prevent an increase in acid concentration. The precipitated lignin was filtered using a glass fiber filter under vacuum filtration and washed with 500 ml water to remove the water-soluble part of the extract generating an ocularly clear filtrate. The fiber residue was washed with water to prevent hydrolysis by the acid from the extraction solvent. Both the lignin and fibers were directly lyophilized after filtration and washing.

To narrow the dispersity of the lignin, ethanol fractionation was performed by mixing lignin and ethanol (1:40 w/w) in a closed vial for 2 hours with magnet stirring. The solution was filtered under vacuum filtration, and the ethanol present in the ethanol-soluble part was removed under reduced pressure by rotary evaporation.

The one-step cyclic extraction of lignin (see STAR Methods) was performed according to the conditions provided in the table below.

The number of cycles and temperatures used to obtain the five different lignins. The lignins are referred to by their abbreviations within this study. The lignins were obtained from the wood species spruce and birch.

**Table:** 

Number of cycles	Abbreviation	Temperature [°C]	Wood species
4	4C	140	Spruce
9	9C	140	Spruce
4 + 4	4C+4C	140 + 160	Spruce
15	15C	160	Spruce
9	9C	140	Birch

#### NMR

The NMR spectroscopy analysis was performed using a Bruker NMR spectrometer Avance III HD 400 MHz (Bruker Corporation, Billerica, MA, USA) instrument. The instrument was equipped with a 5 mm Z-gradient BBFO broadband smart probe (Bruker Corporation, Billerica, MA, USA). The collected NMR data were further processed using MestReNova (v.9.0.0, Mestrelab Research).

The samples for the 2D-NMR HSQC analysis were prepared by dissolving 80 mg lignin in 600 μl DMSO-d6. The pulse program "hsqcetgpsi" was used to acquire the spectra, using 80 scans, a relaxation delay of 1.5 s, and an acquisition time of 0.11 s at a temperature of 297 K, using 1024 × 256 increments. The data were processed using a 90°-shifted square sine-bell apodization window and 1024 × 1024 data points in the software MestReNova. The data were Fourier transformed, and the baseline was corrected in both the ^1^H and ^13^C dimensions by a Bernstein polynomial fit of the third order.

The ^31^P NMR samples were prepared based on previously reported methods,^37^^,^^38^ by dissolving 30 mg of lignin in equal volumes of *N,N*-dimethylformamide (100 μl) and pyridine (100 μl). After dissolution, 50 μl of an internal standard solution (60 mg ml^−1^ eHNDI) in pyridine, 5 mg ml−1 Cr(AcAc3) relaxing agent) was added. Finally, 100 μl of phosphorylating agent, Cl-TMDP, was added to the mixture, followed by the dropwise addition of 450 μl CDCl3. The pulse program "zgig30" was used to acquire the spectra, using 256 scans, a relaxation delay of 5 s, and an acquisition time of 1.68 s at a temperature of 297 K. The data were processed in MestReNova. The data was Fourier transformed, and a Bernstein polynomial fit of the third order corrected the baseline line.

For the ^13^C NMR analysis, 80 mg lignin was dissolved in 600 μl DMSO-d6, with an addition of 0.01 M chromium(III) acetylacetonate (Cr(AcAc3), used as a relaxation agent. The pulse program "sd_zgig90" was used to acquire the spectra, using 26 000 scans, a relaxation delay of 1.7 s, and an acquisition time of 1.36 s at a temperature of 297 K.

#### Size exclusion chromatography

Size exclusion chromatography was carried out on a Waters instrument system (Waters Milford, MA, USA). The setup of the system consisted of an autosampler (2707), HPLC pump (515), and a photodiode array detector (2998) operated at 254 and 280 nm. The separation was performed by the column system of Waters Styragel Guard column (4.6 × 300 mm) in series with Waters Ultrastyragel HR4, HR2 and HR0.5 (4.6 × 300 mm) solvent-efficient GPC columns packed with 5 μm polymer particles. The calibration was performed using polystyrene standards at 254 nm with the nominal molecular weights ranging from 162 to 176 000 Da (specifically, 176 000, 116 000, 46 400, 18 000, 9600, 6540, 2920, 890, 578, 474, 370, 266 and 162 Da). The data acquisition and processing were performed using the Waters Empower 3 build 3471 software.

The average molecular weight distribution and dispersity were evaluated by size exclusion chromatography (SEC) using tetrahydrofuran (THF) as an eluent. To increase the solubility of the lignin in THF, the lignin was acetylated prior to analysis based on a previously published method^39^ by dissolving 2.5 mg of lignin in equal volumes of acetic anhydride (100 μl) and pyridine (100 μl). The sample mixture was stirred overnight at 400 rpm at ambient temperature. To the mixture, an equal volume of cold toluene and methanol was added dropwise during the evaporation of the solvents using a stream of nitrogen until dryness. The residue was dissolved in 1 ml of THF. Prior to analysis, the samples were filtered using a 0.2 μm NYL syringe filter. The separation was performed at a flow rate of 0.3 ml min^−1^. The injection volume was 20 μl, and the separation was performed using a column temperature of 35°C.

#### Carbohydrate analysis

Carbohydrate analysis was performed using an HPAEC/PADICS-3000 system (Thermo Fisher Scientific, Sunnyvale, CA, USA) with pulsed amperometric detection (HPAEC/PAD). The system was equipped with the anion-exchange CarboPac PA-1 (Thermo Fisher Scientific, Sunnyvale, CA, USA) column (4 × 250 mm) with pellicular resin packing. The data were processed using Chromeleon 7.1 (Thermo Fisher Scientific, Sunnyvale, CA, USA) software.

The acid hydrolysis protocol (SCAN-CM 71:09) was followed to quantify the carbohydrate contents in the spruce wood and fiber residue fractions,^40^ where 3 ml of 72% sulfuric acid was added to 200 mg of the 40-mesh milled wood and fiber residue fractions, respectively. This was followed by mixing and vacuum incubation using a desiccator with occasional stirring. After 80 minutes, 84 ml of Milli-Q water was added to the mixture, and the samples were placed in an autoclave for 60 minutes at 125°C. The insoluble residue was thereafter filtered under a vacuum, and the soluble part, mainly containing carbohydrates, was collected and diluted for analysis. The analysis of the carbohydrate composition was performed using an HPAEC/PAD system. The method has been reported elsewhere.^41^ The system was equilibrated using 170 mM sodium acetate and 260 mM sodium hydroxide for 7 minutes, followed by equilibration with Milli-Q water for 6 minutes. Milli-Q was used as a mobile phase at a flow rate of 1 ml min^−1^. At the column effluent before the PAD cell, 300 mM sodium hydroxide was added with a flow rate of 0.5 ml min^−1^. The sugar amounts were estimated from the area of the integrals.

## Data Availability

• All data is available in the main text and in the supplemental information. • All original spectra are available in the supplemental information. • Any additional information required to reanalyze the data reported in this paper is available from the lead contact upon request.

## References

[1] Bruijnincx P.C.A., Weckhuysen B.M. (2014). Lignin up for break-down. Nat. Chem..

[2] Adler E. (1977). Lignin chemistry—past, present and future. Wood Sci. Technol..

[3] White R.H. (1987). Effect of lignin content and extractives on the higher heating value of wood. Wood Fiber Sci..

[4] Ragauskas A.J., Beckham G.T., Biddy M.J., Chandra R., Chen F., Davis M.F., Davison B.H., Dixon R.A., Gilna P., Keller M. (2014). Lignin valorization: improving lignin processing in the biorefinery. Science.

[5] Deuss P.J., Barta K., de Vries J.G. (2014). Homogeneous catalysis for the conversion of biomass and biomass-derived platform chemicals. Catal. Sci. Technol..

[6] Renders T., Van den Bossche G., Vangeel T., Van Aelst K., Sels B. (2019). Reductive catalytic fractionation: state of the art of the lignin-first biorefinery. Curr. Opin. Biotechnol..

[7] Jawerth M., Johansson M., Lundmark S., Gioia C., Lawoko M. (2017). Renewable Thiol–ene thermosets based on refined and selectively allylated industrial lignin. ACS Sustainable Chem. Eng..

[8] Gioia C., Colonna M., Tagami A., Medina L., Sevastyanova O., Berglund L.A., Lawoko M. (2020). Lignin-based epoxy resins: unravelling the relationship between structure and material properties. Biomacromolecules.

[9] Gillet S., Aguedo M., Petitjean L., Morais A.R.C., da Costa Lopes A.M., Łukasik R.M., Anastas P.T. (2017). Lignin transformations for high value applications: towards targeted modifications using green chemistry. Green Chem..

[10] Crestini C., Lange H., Sette M., Argyropoulos D.S. (2017). On the structure of softwood kraft lignin. Green Chem..

[11] Giummarella N., Lindén P.r.A., Areskogh D., Lawoko M., Lindén P.A., Areskogh D., Lawoko M. (2019). Fractional profiling of kraft lignin structure: Unravelling insights on lignin reaction mechanisms. ACS Sustainable Chem. Eng..

[12] Lora J.H., Glasser W.G. (2002). Recent industrial applications of lignin: a sustainable alternative to nonrenewable materials. J. Polym. Environ..

[13] Pye E.K., Lora J.H. (1991). The AlcellTM process: a proven alternative to kraft pulping. Tappi J..

[14] Kleinert T.N. (1971).

[15] Liu Y., Carriero S., Pye K., Argyropoulos D.S. (2000).

[16] Shimada K., Hosoya S., Ikeda T. (1997). Condensation reactions of softwood and hardwood lignin model compounds under organic acid cooking conditions. J. Wood Chem. Technol..

[17] Berlin A., Balakshin M. (2014). Industrial lignins: analysis, properties, and applications. Bioenergy Res, Adv. Appl..

[18] Luo X., Li Y., Gupta N.K., Sels B., Ralph J., Shuai L. (2020). Protection strategies enable selective conversion of biomass. Angew. Chem., Int. Ed..

[19] Shuai L., Amiri M.T., Questell-Santiago Y.M., Héroguel F., Li Y., Kim H., Meilan R., Chapple C., Ralph J., Luterbacher J.S. (2016). Formaldehyde stabilization facilitates lignin monomer production during biomass depolymerization. Science.

[20] Vendamme R., Behaghel de Bueren J., Gracia-Vitoria J., Isnard F., Mulunda M.M., Ortiz P., Wadekar M., Vanbroekhoven K., Wegmann C., Buser R. (2020). Aldehyde-assisted lignocellulose fractionation provides unique lignin oligomers for the design of tunable polyurethane bioresins. Biomacromolecules.

[21] Lan W., Amiri M.T., Hunston C.M., Luterbacher J.S. (2018). Protection group effects during α, γ-diol lignin stabilization promote high-selectivity monomer production. Angew. Chem..

[22] Zijlstra D.S., de Korte J., de Vries E.P.C., Hameleers L., Wilbers E., Jurak E., Deuss P.J. (2021). Highly efficient semi-continuous extraction and in-line purification of high β-O-4 butanosolv lignin. Front. Chem..

[23] Karlsson M., Giummarella N., Lindén P.A., Lawoko M. (2020). Toward a consolidated lignin biorefinery: preserving the lignin structure through additive-free protection strategies. ChemSusChem.

[24] Karlsson M., Vegunta V.L., Deshpande R., Lawoko M. (2022). Protected lignin biorefining through cyclic extraction: gaining fundamental insights into the tuneable properties of lignin by chemometrics. Green Chem..

[25] Sette M., Wechselberger R., Crestini C. (2011). Elucidation of lignin structure by quantitative 2D NMR. Chem. Eur J..

[26] ZHANG L., GELLERSTEDT G. (2000). Sixth European workshop on lignocellulosics and pulp: advances in lignocellulosics chemistry towards high quality processes and products.

[27] Ralph J., Lapierre C., Boerjan W. (2019). Lignin structure and its engineering. Curr. Opin. Biotechnol..

[28] Lourencon T.V., Greca L.G., Tarasov D., Borrega M., Tamminen T., Rojas O.J., Balakshin M.Y. (2020). Lignin-first integrated hydrothermal treatment (HTT) and synthesis of low-cost biorefinery particles. ACS Sustainable Chem. Eng..

[29] Giummarella N., Lawoko M. (2017). Structural insights on recalcitrance during hydrothermal hemicellulose extraction from wood. ACS Sustainable Chem. Eng..

[30] Samuel R., Cao S., Das B.K., Hu F., Pu Y., Ragauskas A.J. (2013). Investigation of the fate of poplar lignin during autohydrolysis pretreatment to understand the biomass recalcitrance. RSC Adv..

[31] Trajano H.L., Engle N.L., Foston M., Ragauskas A.J., Tschaplinski T.J., Wyman C.E. (2013). The fate of lignin during hydrothermal pretreatment. Biotechnol. Biofuels.

[32] Bauer S., Sorek H., Mitchell V.D., Ibáñez A.B., Wemmer D.E. (2012). Characterization of Miscanthus giganteus lignin isolated by ethanol organosolv process under reflux condition. J. Agric. Food Chem..

[33] Fisher H.E., Kulka M., Hibbert H. (1944). Studies on lignin and related compounds. LXXIX. Synthesis and properties of 3-Hydroxy-1-(3, 4-dimethoxyphenyl)-2-propanone1. J. Am. Chem. Soc..

[34] Miles-Barrett D.M., Neal A.R., Hand C., Montgomery J.R.D., Panovic I., Ojo O.S., Lancefield C.S., Cordes D.B., Slawin A.M.Z., Lebl T., Westwood N.J. (2016). The synthesis and analysis of lignin-bound Hibbert ketone structures in technical lignins. Org. Biomol. Chem..

[35] Vanholme R., Morreel K., Darrah C., Oyarce P., Grabber J.H., Ralph J., Boerjan W. (2012). Metabolic engineering of novel lignin in biomass crops. New Phytol..

[36] Balakshin M., Capanema E.A., Zhu X., Sulaeva I., Potthast A., Rosenau T., Rojas O.J. (2020). Spruce milled wood lignin: linear, branched or cross-linked?. Green Chem..

[37] Argyropoulos D.S. (1995). 31 P NMR in wood chemistry: a review of recent progress. Res. Chem. Intermed..

[38] Granata A., Argyropoulos D.S. (1995). 2-Chloro-4, 4, 5, 5-tetramethyl-1, 3, 2-dioxaphospholane, a reagent for the accurate determination of the uncondensed and condensed phenolic moieties in lignins. J. Agric. Food Chem..

[39] Gellerstedt G. (1992). Methods in Lignin Chemistry.

[40] SCAN (2009).

[41] Azhar S., Henriksson G., Theliander H., Lindström M.E. (2015). Extraction of hemicelluloses from fiberized spruce wood. Carbohydr. Polym..

