# Supplementary material for: A flexible physical protection process for lignin extraction
Source: iScience. 2023 Jul 28;26(9):107507. doi: 10.1016/j.isci.2023.107507 (PMC10448533; doi:10.1016/j.isci.2023.107507)
Supplement: Document S1. Figures S1–S13 and Tables S1–S5 [file mmc1.pdf]

iScience, Volume *26*

Supplemental information

A flexible physical protection process for lignin extraction

Maria Karlsson and Martin Lawoko

Supporting information

A Flexible Physical Protection Process for Lignin Extraction

Maria Karlsson,*[1,2] and Martin Lawoko *[a,b]

[1] Wallenberg Wood Science Center, Department of Fiber and Polymer Technology, School of Chemistry, Biotechnology and Health, KTH Royal Institute of Technology, Teknikringen 56-58, SE-100 44 Stockholm, Sweden.

[2] Division of Wood Chemistry and Pulp Technology, Department of Fiber and Polymer Technology, School of Chemistry, Biotechnology and Health, KTH Royal Institute of Technology, Teknikringen 56-58, SE-100 44 Stockholm, Sweden.

* Corresponding authors: maria11@kth.se (M.K.), lawoko@kth.se (M.L.)

Contents

1. HSQC NMR 2
  1.1 Assigned of diagnostic chemical shifts for HSQC NMR integration 2
  1.2 Quantification of interunit linkages 2
  1.1 HSQC NMR spectra 3
2. ^{31}P NMR 7
  2.1 Assignment of diagnostic chemical shifts for lignin hydroxyl functionalities. 7
  2.2 Quantification of hydroxyl functionalities. 7
  2.4 ^{31}P NMR spectra 8
3. ^{13}C NMR spectra 10
4. Size exclusion chromatography 11
5. Water-soluble fractions 12
References 13

1. HSQC NMR

1.1 Assigned of diagnostic chemical shifts for HSQC NMR integration

Table S1. Chemical shift for assignment of spruce and birch lignin, related to Figure 2 and 4.

Shift (ppm)	C2[1]	C2[2]	β-O-4´$_{\alpha}$	β-O-4´$_{Et,\alpha}$	β-O-4´G$_{\beta}$[1]	β-O-S4´$_{\beta}$[2]	DBDO $_{\beta}$	β-5´$_{\alpha}$	β- β´$_{\alpha}$	β-1´, stilbene$_{\alpha}$	β-5´, stilbene$_{\beta}$	Coumaryl-aldehyde$_{\alpha}$	HK$_{\gamma}$
^{1}H	7.6-6.3	6.9-6.1	5.5-4.5	4.8-4.3	4.6-4.1	4.2-3.9	4.0-3.7	5.9-5.2	4.8-4.5	7.1-6.9	7.4-7.17	7.8-7.5	4.3-4.1
^{13}C	113.2-106.5	109.8-99.7	74.7-67.2	81.1-76.9	87.2-80.9	88.0-83.0	87.4-83.8	90.2-83.4	86.0-83.1	128.4-124.1	121.4-117.4	156.6-149.5	68.7-65.1

[1]The shifts are specifically for G-related unit. [2]The shifts are specifically for S-related unit.

1.2 Quantification of interunit linkages

Table S2. Quantification of interunit linkages for birch lignin, related to Figure 4.

Lignin	β-O-4´$_{\alpha}$	β-5´ $_{\alpha}$	β- β´$_{\alpha}$	CA$_{\alpha}$[1]	HK$_{\gamma}$[2]
			per 100 Ar		
Birch, 9C, 140 °C	67	4.1	5.6	0.81	0.73

[1]CA = Coumaryl-aldehyde. [2]HK=Hibberts's ketone.

1.1 HSQC NMR spectra

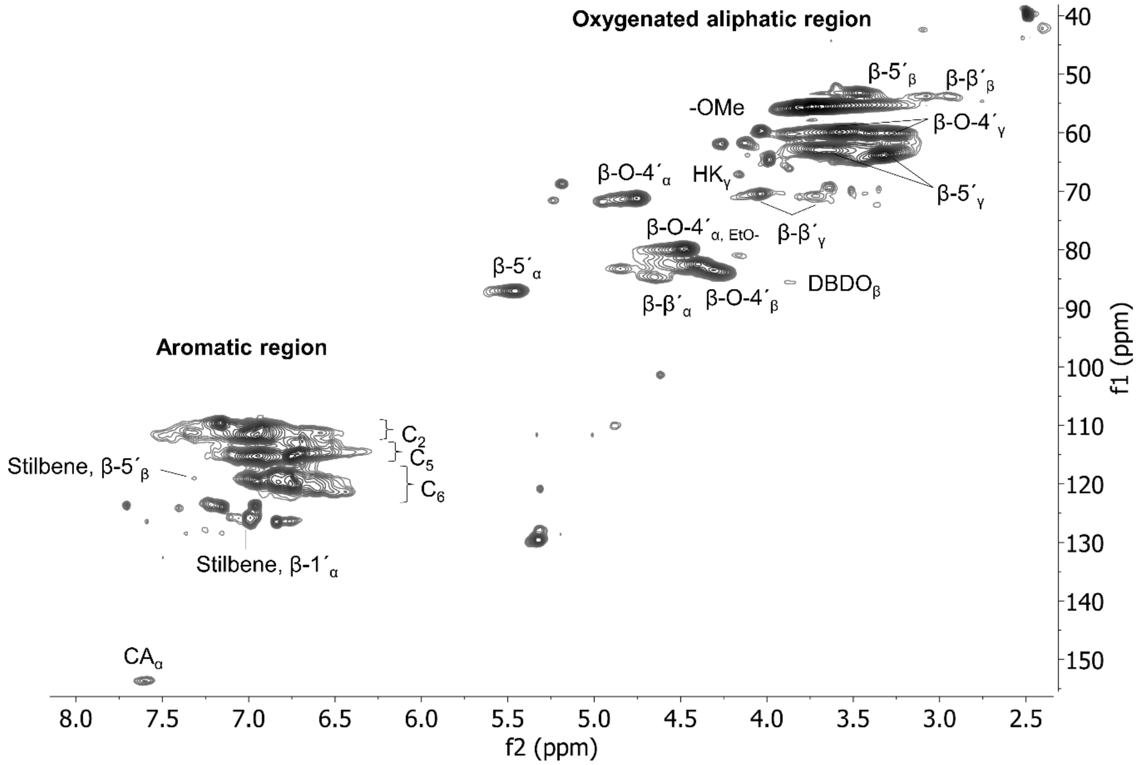

Figure S1. HSQC NMR spectra of spruce lignin obtained using 4C, 140 °C, where f1 corresponds to the ^{13}C dimension and f2 to the ^{1}H dimension, related to Figure 2.

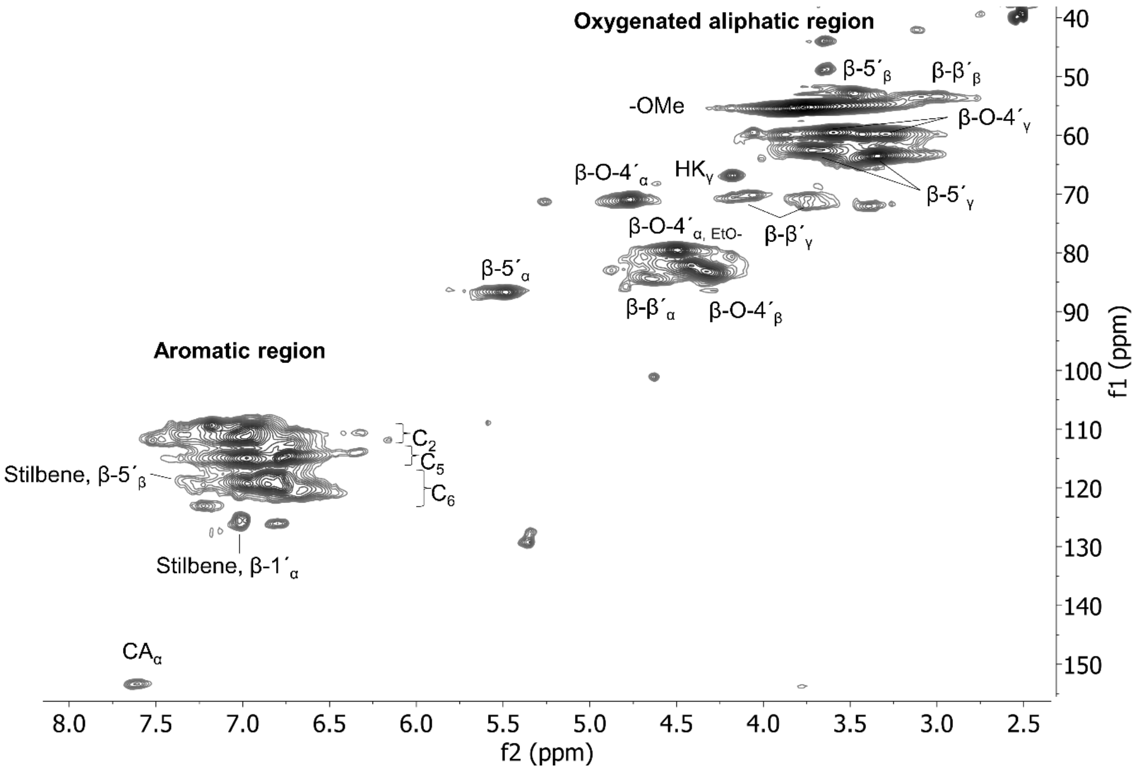

Figure S2. HSQC NMR spectra of spruce lignin obtained using 4C+4C, 140 °C followed by 160 °C, where f1 corresponds to the ^{13}C dimension and f2 to the ^{1}H dimension, related to Figure 2.

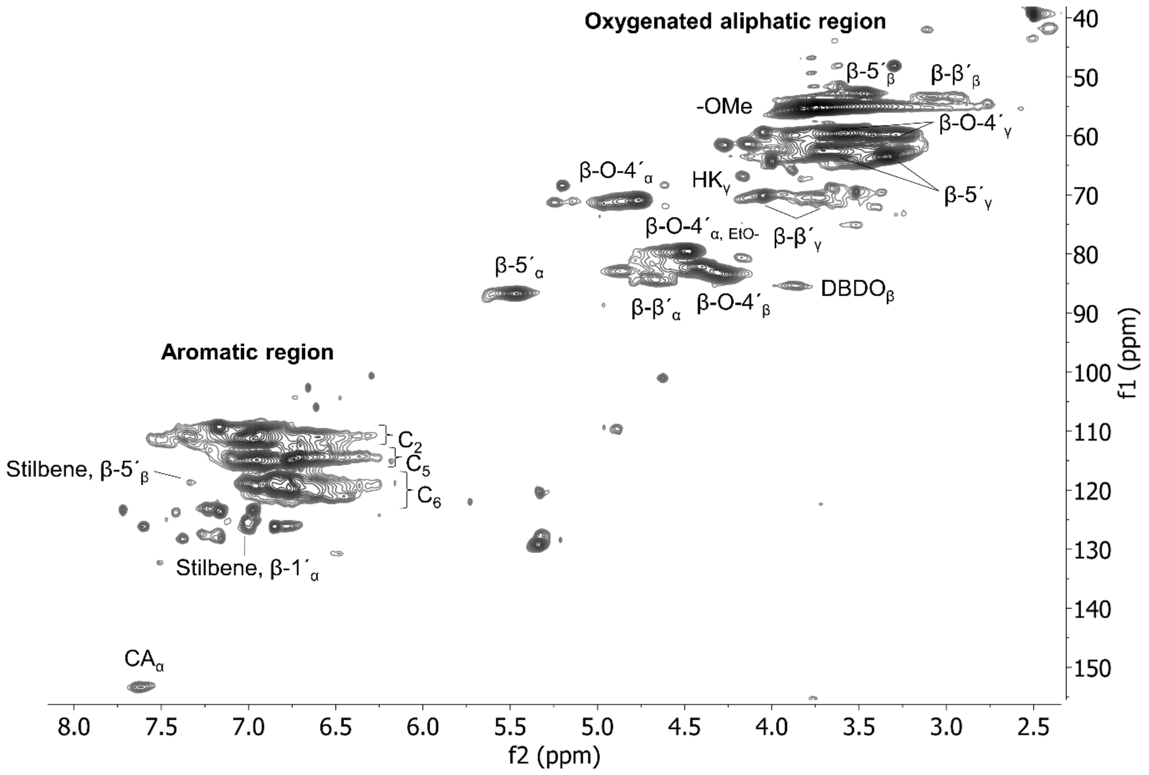

Figure S3. HSQC NMR spectra of spruce lignin obtained using 9C, 140 °C, where f1 corresponds to the ^{13}C dimension and f2 to the ^{1}H dimension, related to Figure 2.

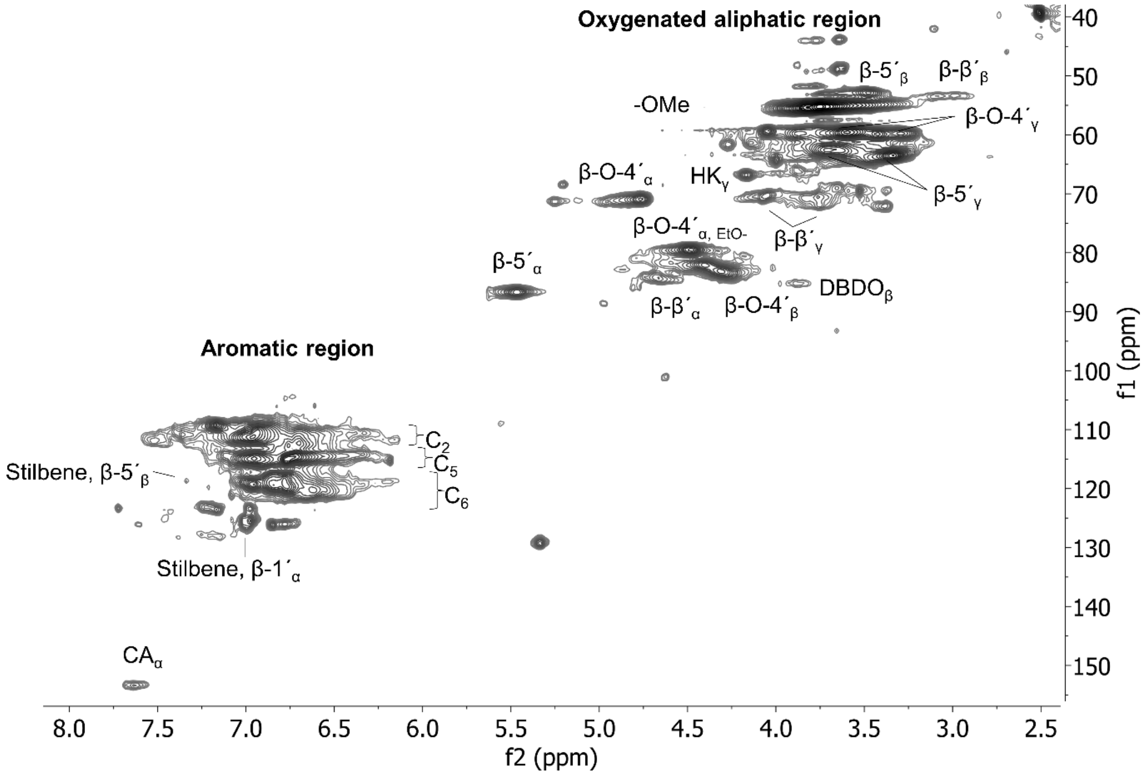

Figure S4. HSQC NMR spectra of spruce lignin obtained using 15C, 160 °C, where f1 corresponds to the ^{13}C dimension and f2 to the ^{1}H dimension, related to Figure 6.

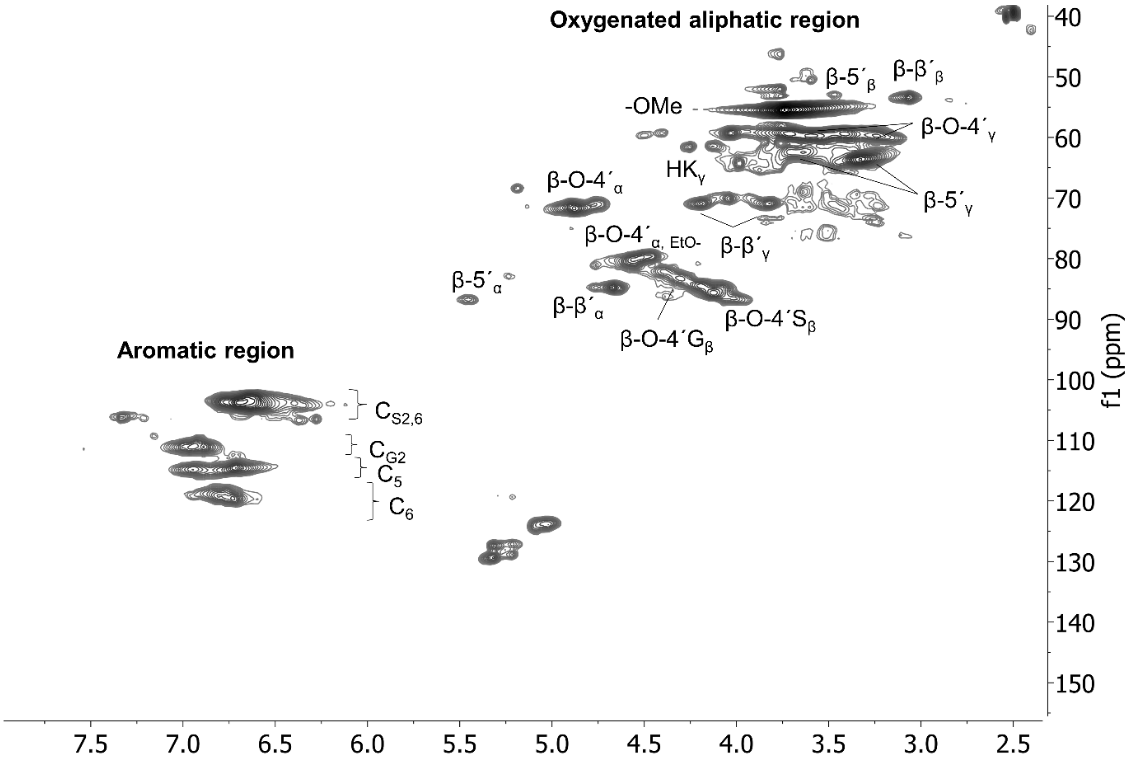

Figure S5. HSQC NMR spectra of birch lignin obtained using 9C, 140 °C, where f1 corresponds to the ^{13}C dimension and f2 to the ^{1}H dimension, related to Figure 4.

2. ^{31}P NMR

2.1 Assignment of diagnostic chemical shifts for lignin hydroxyl functionalities.

Table S3. The chemical shifts (ppm) are relative to the water reaction product of Cl-TMDP at 132.2 ppm, related to Figure 3.

Hydroxyl functionality	***e*HNDI**	**Aliphatic-OH**	**Condensed-OH**	**Non-condensed-OH**		**Carboxylic acids-OH**
			**β5, 4-O-5, 5-5**	**Guaiacyl-OH**	***p*-hydroxy phenyl-OH**	
Chemical shift	152.3-151.7	149.1-145.1	144.7-141.1	140.6-138.8	138.2-137.3	136.6-133.6

2.2 Quantification of hydroxyl functionalities.

Table S4. Quantification of hydroxyl functionalities for spruce and birch lignin, related to Figure 3.

**Lignin**	**Aliphatic-OH**	**C_5 substituted-OH**	**Guaiacyl-OH**	**p-hydroxy phenyl-OH**	**Carboxylic acid-OH**
			mmol/g		
Spruce, 4C, 140 °C	3.5	0.27	0.96	0.046	0.25
Spruce, 4C, 160 °C	3.5	0.55	0.96	0.046	0.051
Spruce, 9C, 140 °C	3.8	0.32	0.93	0.080	0.27
Spruce, 15C, 160 °C	n.a.[1]	n.a.	n.a.	n.a.	n.a.
Birch, 9C, 140 °C	4.4[2]		0.55	0.074	0.17
Alcell, 160 °C[3]	1.4	1.2	1.5	0.18	0.33

[1]n.a.= not analyzed. [2]Aliphatic-OH and C_5 substituted-OH integrated together due to sinapyl-OH overlapping. [3]The values are collected from previous work. A 2 h batch extraction at 160 °C was performed using the same extraction solvent presented within the present study.[1]

2.4 ^{31}P NMR spectra

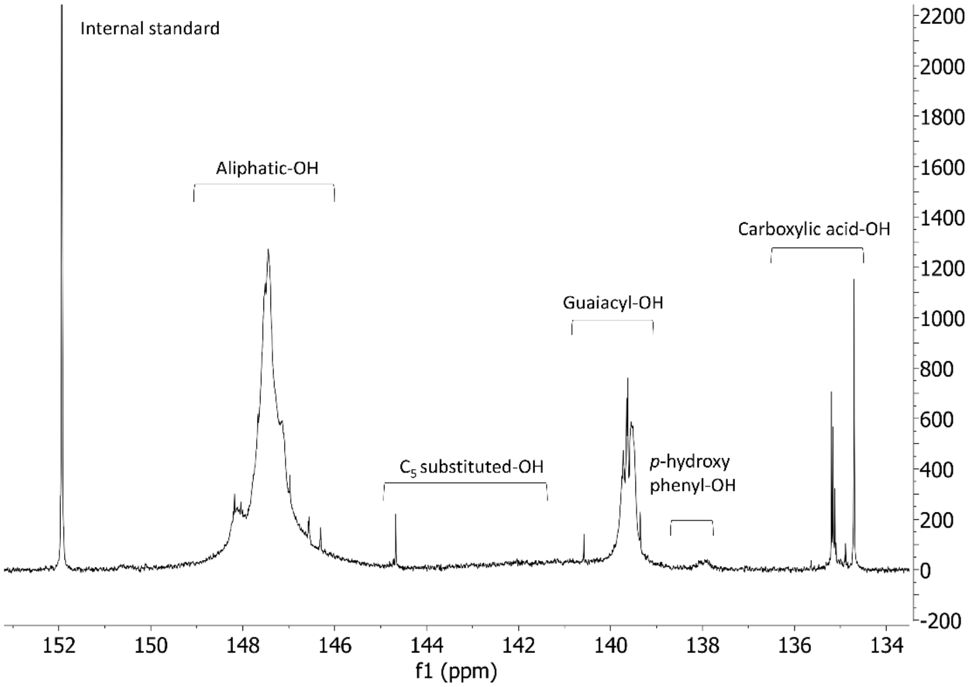

Figure S6. ^{31}P NMR spectra of spruce lignin obtained using 4C at 140 °C, related to Figure 3.

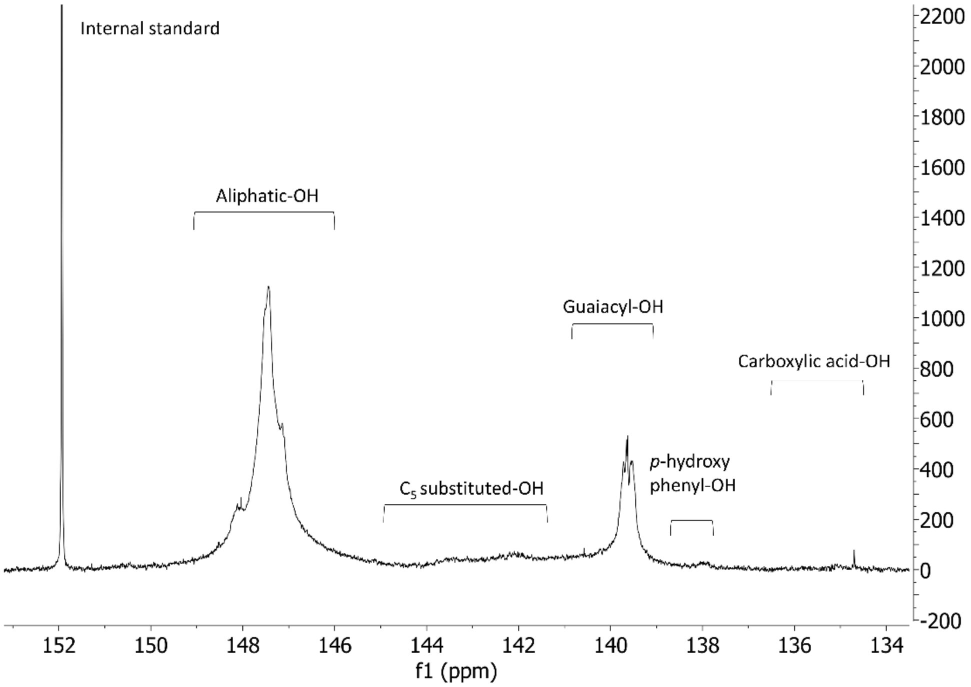

Figure S7. ^{31}P NMR spectra of spruce lignin obtained using 4C+4C, 140 °C followed by 160 °C, related to Figure 3.

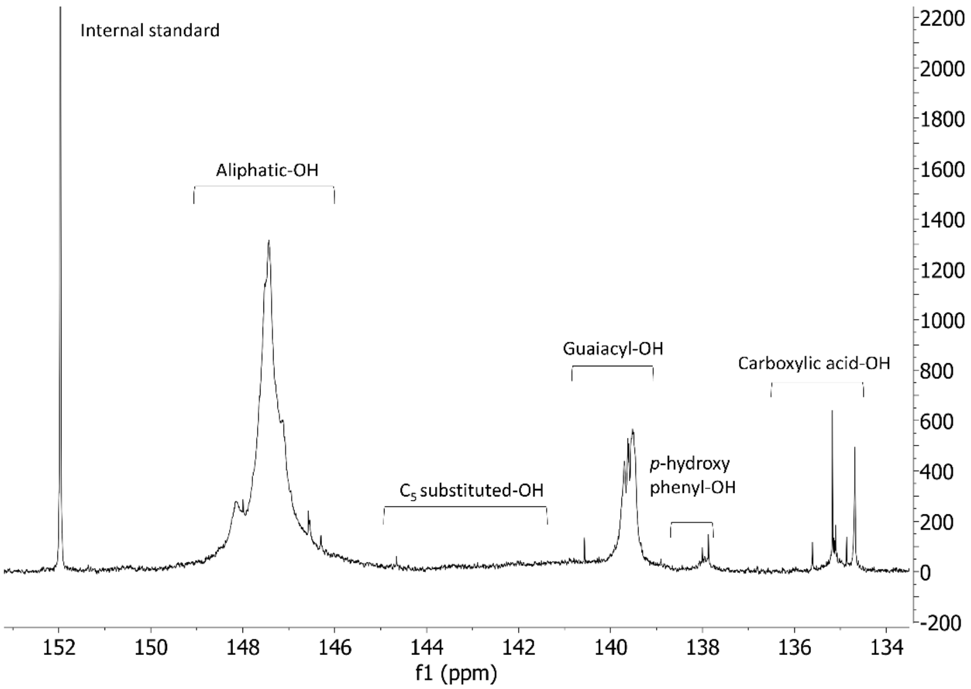

Figure S8. ^{31}P NMR spectra of spruce lignin obtained using 9C at 140 °C, related to Figure 3.

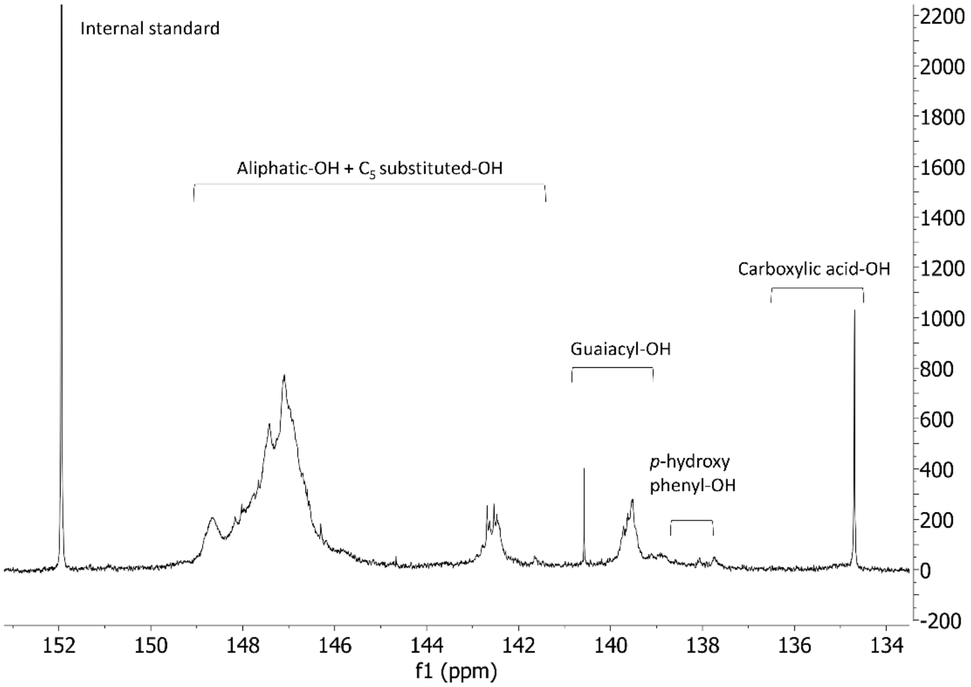

Figure S9. ^{31}P NMR spectra of birch lignin obtained using 9C at 140 °C, related to Figure 3.

3. ^{13}C NMR spectra

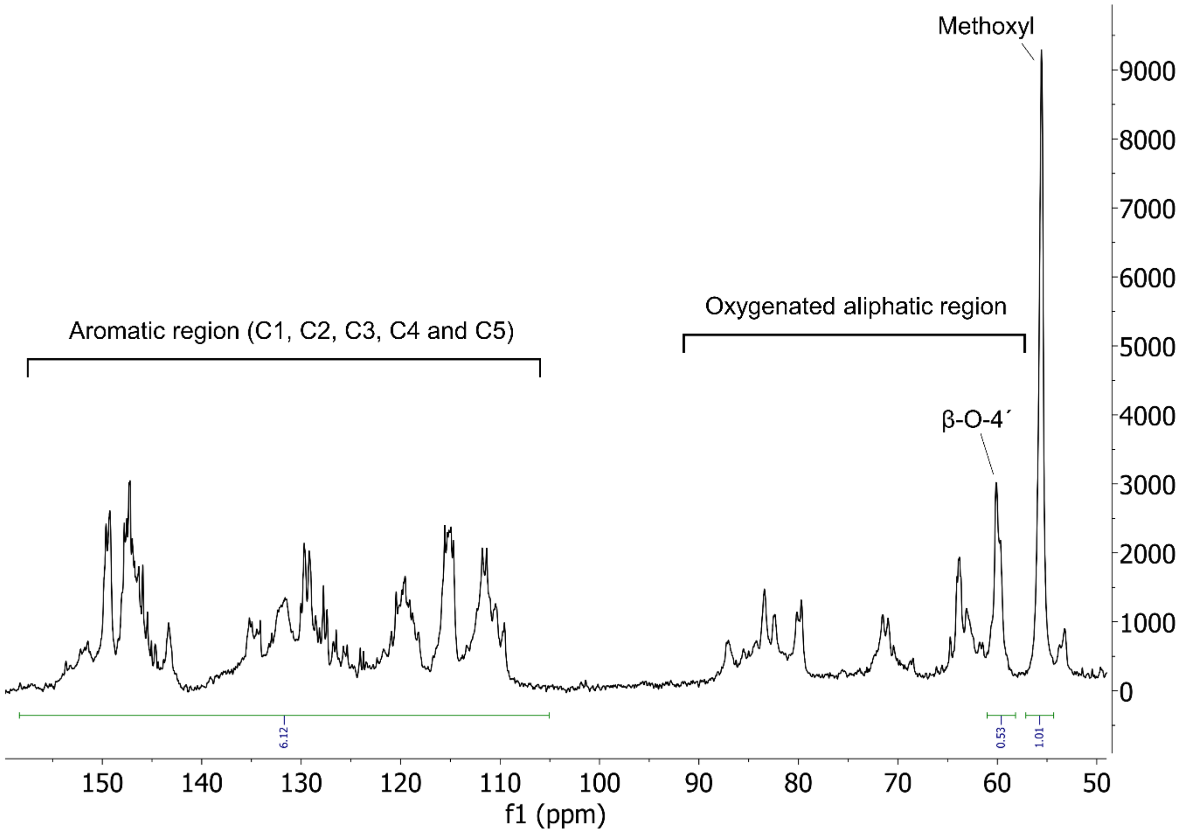

Figure S10. ^{13}C NMR spectra of lignin obtained using 4 cycles at 140 °C including quantification of β-O-4´ interunit linkages, related to "Results and discussion" in the main text.

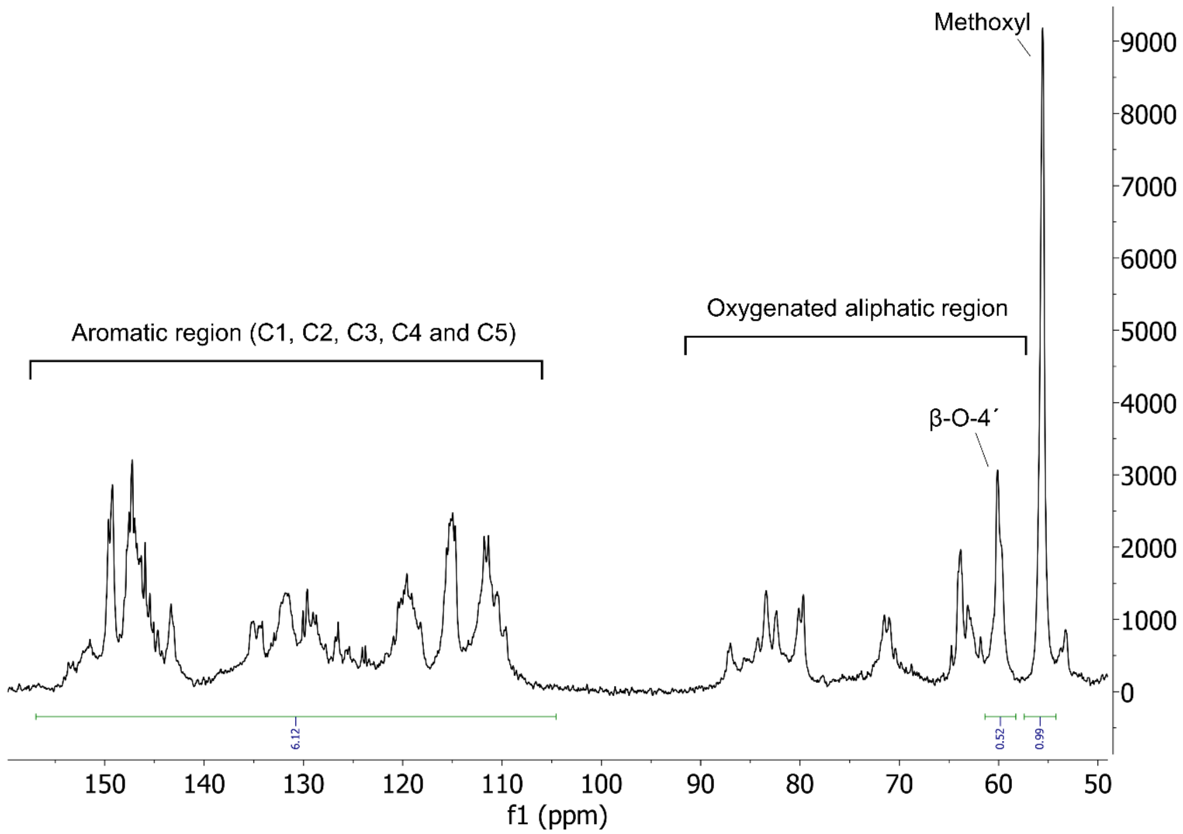

Figure S11. ^{13}C NMR spectra of lignin obtained using 9 cycles at 140 °C including quantification of β-O-4´ interunit linkages, related to "Results and discussion" in the main text.

4. Size exclusion chromatography

Table S5. The molecular weight distribution (Mn), dispersity (Đ) and degree of polymerization (DP_n) of the lignin fractions, related to Figure 5.

Lignin	Mn	Đ	DP_n
Spruce, 4C, 140 °C	2200 ± 30[2]	4.6 ± 0.04	12
Spruce, 4C, 160 °C[1]	3400 ± 20	4.7 ± 0.03	19
Spruce, 9C, 140 °C	2400 ± 20	6.0 ± 0.03	13
Spruce, 4C, 140 °C, EtOH soluble	1300 ± 30	2.3 ± 0.03	7
Spruce, 4C, 140 °C, EtOH insoluble	6600 ± 550	2.2 ± 0.09	36
Birch, 9C, 140 °C	2500 ± 30	4.1 ± 0.03	13

[1]4 cycle extraction immediately after 4 cycle extraction at 140 °C. [2]Number of replicates, n=2.

5. Water-soluble fractions

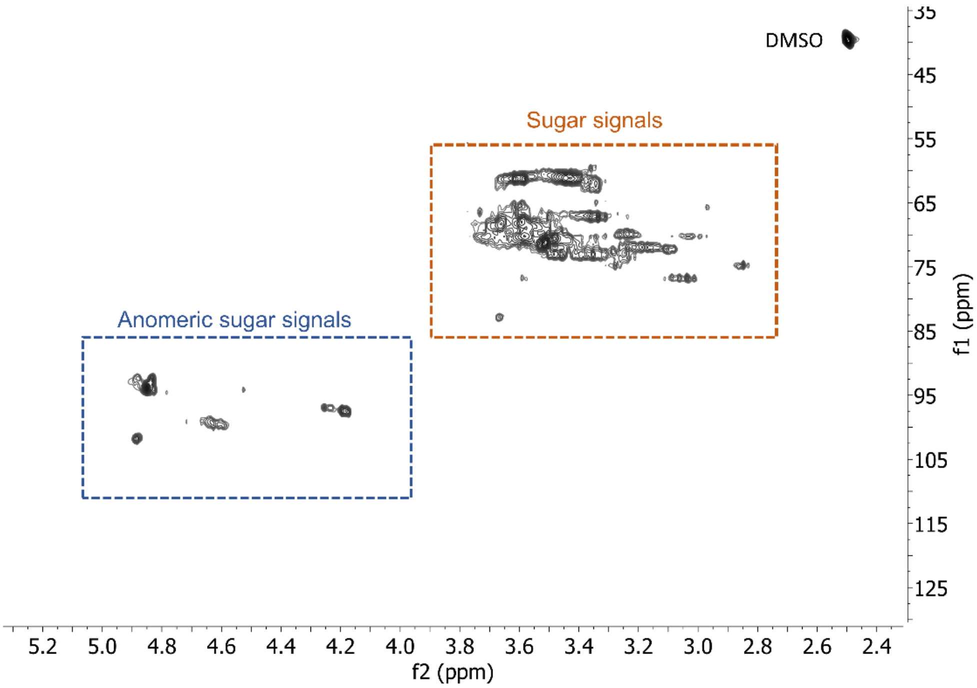

Figure S12. HSQC NMR spectra of the water-soluble fraction, 4C, 140 °C, where f1 corresponds to the ^{13}C dimension and f2 to the ^{1}H dimension, related to "Results and discussion" in the main text.

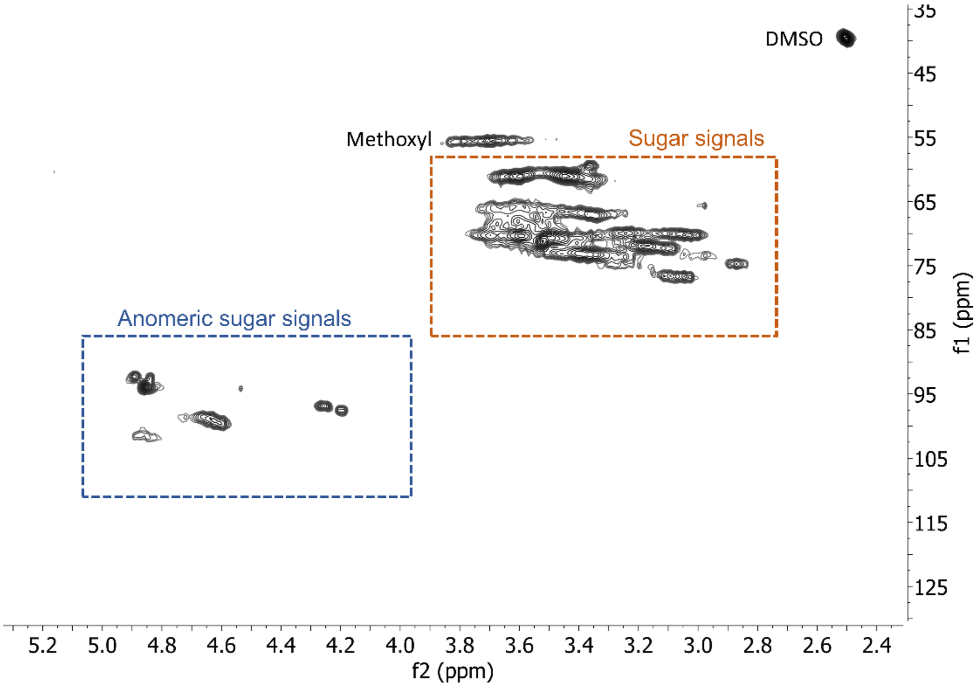

Figure S13. HSQC NMR spectra of the water-soluble fraction, 15C, 160 °C, where f1 corresponds to the ^{13}C dimension and f2 to the ^{1}H dimension, related to "Results and discussion" in the main text.

References

1. Karlsson, M., Vegunta, V.L., Deshpande, R., and Lawoko, M. (2022). Protected lignin biorefining through cyclic extraction: gaining fundamental insights into the tuneable properties of lignin by chemometrics. Green Chem. *24*, 1211-1223, 10.1039/D1GC04171A.
